# A TSC22-like motif defines a novel antiapoptotic protein family

**DOI:** 10.1111/j.1567-1364.2008.00367.x

**Published:** 2008-03-18

**Authors:** Chamel M Khoury, Zhao Yang, Xiao Yu Li, Marissa Vignali, Stanley Fields, Michael T Greenwood

**Affiliations:** 1 Department of Medicine, McGill UniversityMontreal, Quebec, Canada; 2 Department of Genome Sciences, University of WashingtonSeattle, WA, USA; 3 Howard Hughes Medical Institute, University of WashingtonSeattle, WA, USA; 4 Department of Chemistry and Chemical Engineering, Royal Military College (RMC)Kingston, ON, Canada

**Keywords:** TSC22, yeast, leucine zipper, antiapoptosis, FYV10, SNO1

## Abstract

The apoptotic programme is evolutionarily conserved between yeast and metazoan organisms. We have previously identified a number of mammalian cDNAs capable of suppressing the deleterious effects of Bax expression in yeast. We herein report that one such suppressor, named Tsc22^(86)^, represents the C-terminal 86 amino acids of the previously characterized leucine zipper (LZ) motif-containing transcriptional regulator Tsc22. Employing a genome-wide two-hybrid screen, functional genomics, and deletion mutagenesis approaches, we conclude that Tsc22^(86)^-mediated antiapoptosis is independent of the LZ motif and is likely independent of effects on gene transcription. Rather, a 16-residue sequence within the conserved 56-residue TSC22 domain is necessary for antiapoptosis. The presence of a similar sequence was used to predict an antiapoptotic role for two yeast proteins, Sno1p and Fyv10p. Overexpression and knock-out experiments were used to validate this prediction. These findings demonstrate the potential of studying heterologous proteins in yeast to uncover novel biological insights into the regulation of apoptosis.

## Introduction

Understanding the basic mechanisms of mammalian apoptosis has been facilitated by the genetic work done in the metazoan worm *Caenorhabditis elegans* (Lettre & Hengartner, 2006). Unicellular organisms, such as the yeast *Saccharomyces cerevisiae*, have also been shown to undergo apoptotic-like programmed cell death (CD). Although initially controversial, the large and ever increasing body of literature makes it quite clear that yeast undergoes a form of PCD that is similar to the process of mitochondrial or intrinsic apoptotic cell death that occurs in mammalian cells (Madeo *et al.*, 1999; Gourlay & Ayscough, 2005; Frohlich *et al.*, 2007). Yeast undergoing apoptotic-like cell death show the typical hallmarks of a mammalian apoptotic cell such as DNA cleavage, chromatin condensation and externalization of phosphatidylserine, as well as an elevated level of reactive oxygen species (ROS). These similarities are further evident by the discovery that yeast apoptosis is also regulated by a number of different proteins that have been shown to be important in mammalian apoptosis. Yeast possess an AIF (Apoptosis Inducing Factor; *AIF*), an AMID (AIF-homologous Mitochondrion-associated Inducer of Death; *NDI1*), a caspase (*YCA1*), an IAP (inhibitor of apoptosis; *BIR1*), an Omi/Htr2A (*NMA111*), a DJ-1 (*HSP31*), as well as a nuclease (*TAT-D*) that is a strong candidate to be involved cell death mediated DNA degradation (Frohlich *et al.*, 2007; Skoneczna *et al.*, 2007). Analyses of many of these proteins show that they perform similar functions as their metazoan counterparts. For example, it was recently reported that the yeast ortholog of the Omi/Htr2A serine protease (Nma111p) induces apoptosis in part by cleaving yeast IAP Bir1p (Walter *et al.*, 2006). Furthermore, overexpression of the yeast pro-apoptotic proteins can serve to initiate or enhance cell death while yeast strains lacking any of these genes show a decrease response to a number of different death stimuli (Madeo *et al.*, 2002; Fahrenkrog *et al.*, 2004). In addition to the extensively studied antiapoptotic regulators, such as antioxidant enzymes and heat shock proteins, cells express a range of molecules that serve to prevent apoptosis and other forms of cell death (Reed, 2004). Given that the biochemical or molecular functions of many antiapoptotic proteins are not well known, it is probable that much remains to be answered regarding the strategies cells use to counter pro-apoptotic mechanism (Reed, 2004). In addition, the response to oxidative stress is far more pleiotropic than commonly described (Temple *et al.*, 2005). For example, as many as 600 different genes have been found to be required for full resistance to oxidative stress while 900 or so genes have been shown to be induced by different stresses (Winzeler *et al.*, 1999; Gasch *et al.*, 2000).

The process of antiapoptosis in yeast shows notable similarities to mammalian cells. For example, MAP kinase signalling cascades play important roles in counteracting the effects of ER stress in yeast and mammalian cells (Hu *et al.*, 2004; Chen *et al.*, 2005). Also, the analysis of the Parkinson's disease associated presynaptic protein α-synuclein has been used as a tool to identify novel proteins that can prevent endoplasmic reticulum (ER) stress-mediated cell death in both yeast and metazoans (Zabrocki *et al.*, 2005; Griffioen *et al.*, 2006; Witt & Flower, 2006). The ability to screen heterologous libraries to identify suppressors of the Bax-mediated cell death in yeast has also served to shed light on the process of antiapoptosis. For example, the identification of ROS scavenging proteins as suppressors of the lethal effects of Bax expression clearly implicates the role of ROS in PCD in yeast (Kampranis *et al.*, 2000; Moon *et al.*, 2002). We have made a concerted effort to study the process of antiapoptosis in yeast through the identification of a number candidate antiapoptotic mammalian cDNAs effective in preventing the inhibitory effects of Bax expression in yeast (Yang *et al.*, 2006; Khoury *et al.*, 2007; Khoury & Greenwood, 2008). The identification of sphingomyelin synthase 1 (SMS1), a ceramide-depleting enzyme, as an antiapoptotic sequence in yeast highlights the central role of sphingolipid metabolism in yeast PCD (Yang *et al.*, 2006).

In a previous report, human TSC22 was identified as one of a number of suppressors of Bax-mediated cell death in yeast (Yang *et al.*, 2006). TSC22 was first identified as a 144-residue leucine zipper (LZ) motif-containing protein that is up-regulated by transforming growth factor-β1 (TGF-β1 (Shibanuma *et al.*, 1992). ‘TSC22’ is now a commonly accepted denotation for a LZ motif-containing conserved domain encoded for by a multi-gene family (Fiol *et al.*, 2007). Although the TSC22 domain is highly conserved between the different TSC22 domain-containing proteins, there appears to be distinct functional differences. For example, some members are proapoptotic while others are distinctly antiapoptotic (Kawamata *et al.*, 2004; Fiol *et al.*, 2007). Although yeast does not have a Tsc22 ortholog, we reasoned that its potential antiapoptotic effects in yeast would likely function by its ability to interact with a LZ transcription factor, reminiscent of the current model for Tsc22 function in mammalian cells (Kester *et al.*, 1999; Kawamata *et al.*, 2004). Here we report that the cDNA encoding the C-terminal TSC22 domain of the human *TSC22-1* gene prevent yeast cell death in response to a variety of apoptotic stimuli including the ROS donor hydrogen peroxide (H_2_O_2_). Global two-hybrid analysis, the analysis of yeast mutants lacking LZ transcription factors, as well as the analysis of TSC22 deletions were used to demonstrate that the LZ structure of TSC22 is not required for this antiapoptotic function. Instead our analysis has lead to the identification of a 16 amino acid (aa) motif that is required to confer protection against ROS in yeast. The 16 aa sequence is present in multiple proteins including four different yeast proteins. The demonstration that two of these proteins, Sno1p and Fyv10p, are indeed antiapoptotic suggests that we have uncovered a new motif that confers antiapoptotic effects.

## Materials and methods

### Yeast strains and plasmids

Strain BY4741 (*MAT***a** his3Δ1 leu2Δ0 met15Δ0 ura3Δ0) was used as the wild type strain. Strain KTY3 (*pep4∷kanMX, prb1∷LEU2,His3MX6-P*_*GAL1*_*-3HA∷ERG6*) contains a genomic insertion of the *GAL1* promoter upstream of the *ERG6* coding sequence. Both this strain and its parental KTY1 (*pep4∷kanMX,act1-157∷HIS3*) were gifts from G. Eitzen. All deletion mutants were isogenic to BY4741 and were obtained from EUROSCARF (http://web.uni-frankfurt.de/fb15/mikro/euroscarf/index.html). The Bax suppressors (Sup. 12, Sup. 32, and Sup. 97) were previously isolated by screening a human heart expression cDNA library in yeast cells expressing Bax under the control of the *GAL1* promoter (Yang *et al.*, 2006). The heart cDNA library and consequently the Bax suppressors used in this study are cloned into the galactose inducible pYES-DEST52 vector. The sequences encoding TSC22-1v2, and TSC22-1v3, as well as the TSC22^(86)^ deletion mutants were amplified by PCR using either a heart cDNA library (Yang *et al.*, 2006) or the cDNA-encoding TSC22^(86)^ as a template. All the primers used: in this study are described in Table 1. The TSC22 domains (and nonconserved C-terminal tails) encoded by *TSC22-2*, *TSC22-3*, and *TSC22-4* were also amplified by PCR using either a human heart or skeletal muscle cDNA library as template. All these PCR products were subcloned into the yeast expression vector p426GAL1. For two hybrid plasmids, the TSC22^(86)^ coding sequence was PCR amplified and cloned by recombination in yeast into the vector pOBD2 (McCraith *et al.*, 2000) in frame with the Gal4 DNA-binding domain (Gal4DBD) (Yang *et al.*, 2007). The TSC22^(86)^ coding sequence was also PCR amplified and cloned by recombination in yeast into the vector pLEXA-dir (McCraith *et al.*, 2000) in frame with the Lex DNA-binding domain (LexADBD). The resulting constructs were confirmed by sequencing. The three LZ-containing yeast Transcription Factor (TFs) (*RTG1, RTG3* and *CST6*) were prepared for subcloning into the two-hybrid prey vector by PCR amplification from a yeast genomic library as a template. The PCR products were subcloned in frame with Gal4 Activation Domain into plasmid pACT2 (Gyuris *et al.*, 1993). The ORFs encoding Sno1p and Fyv10p were amplified by PCR using a yeast genomic library as a template. The PCR products were subcloned in frame with green fluorescent protein (GFP) in vector p426GAL1-GFP (Somerville *et al.*, 2003).

**Table 1:** Sequences of the oligonucleotides used in this study

Name	Oligonucleotides (5′–3′)
Expression	
TSC22-1v2 Forward	TAGTGGATCCCCCGGGCTGCAGGAATTCGAATGGGAGCCCCTACTGTGGTG
TSC22-1v3 Forward	TAGTGGATCCCCCGGGCTGCAGGAATTCGAATGAAATCCCAATGGTGTAGA
TSC22^(86)^ Forward	TAGTGGATCCCCCGGGCTGCAGGAATTCGAATGGATCTAGTGAAAAGCCAT
TSC22-1 Reverse	GGTGGCGATGGATCCCGGGCCCGCGGTACCTGCGGTTGGTCCTGAGCCCTG
TSC22-2 Forward	TAGTGGATCCCCCGGGCTGCAGGAATTCGAATGGATCTGGTGAAAAGCCAT
TSC22-2 Reverse	GGTGGCGATGGATCCCGGGCCCGCGGTACCTTATGCTGAGGAGACATTCGG
TSC22-3 Forward	TAGTGGATCCCCCGGGCTGCAGGAATTCGAATGGATCTGGTGAAGAATCAT
TSC22-3 Reverse	GGTGGCGATGGATCCCGGGCCCGCGGTACCCACCGCAGAACCACCAGGGGC
TSC22-4 Forward	TAGTGGATCCCCCGGGCTGCAGGAATTCGAATGGACTTGGTGAAGTCCCAC
TSC22-4 Reverse	GGTGGCCATGGATCCCGGGCCCGCGGTACCTCAGACGGAGGGCCCATTGGG
TSC22^(Δ1−40)^ Forward	TAGTGGATCCCCCGGGCTGCAGGAATTCGAATGAAGACACTGGCCAGTCCT
TSC22^(Δ1−40)^ Reverse	GGTGGCGATGGATCCCGGGCCCGCGGTACCTGCGGTTGGTCCTGAGCCCTG
TSC22^(Δ1−56)^ Forward	TAGTGGATCCCCCGGGCTGCAGGAATTCGATATGCGGTCAGAGAAGAAGTC
TSC22^(Δ1−56)^ Reverse	GGTGGCGATGGATCCCGGGCCCGCGGTACCTGCGGTTGGTCCTGAGCCCTG
TSC22^(Δ41−86)^ Forward	TAGTGGATCCCCCGGGCTGCAGGAATTCGAATGGATCTAGTGAAAAGCCAT
TSC22^(Δ41−86)^ Reverse	GGT GGCCATGGATCCCGGGCCCGCGGTACCCAGCAGATTGTTGTTCTCCTG
TSC22^(Δ57−86)^ Forward	TAGTGG ATCCCCCGGGCTGCAGGAATTCGAATGGATCTAGTGAAAAGCCAT
TSC22^(Δ57−86)^ Reverse	GGTGGCCATGGATCCCGGGCCCGCGGTACCCTGGGCCTGAAACTGGGCAAG
*SNO1* Forward	TAGTGGATCCCCCGGGCTGCAGGAATTCGAATGCACAAAACCCACAGTACA
*SNO1* Reverse	GGTGGCCATGGATCCCGGGCCCGCGGTACCATTAGAAACAAACTGTCTGAT
*FYV10*Forward	TAGTGGATCCCCCGGGCTGCAGGAATTCGAATGGCAGAGAAATCAATATTT
*FYV10* Reverse	GGTGGCCATGGATCCCGGGCCCGCGGTACCGGTTGGGTACATTTTGATAGA
TSC22^(86)^2byb Forward	CCAAAAAAAGAGATCGAATTCCAGCTGACCATGGATCTAGTGAAAAGCCAT
TSC22^(86)^2byb Reverse	ATCTCTGCAGGTCGACGGATCCCCGGGAATCTATGCGGTTGGTCCTGAGCC
*RTG1* Forward	AGCTTGGGTGGTCATATGGCCATGGAGGCCATGAGCAGCATTCCAGCTGGC
*RTG1* Reverse	GTTTTTCAGTATCTACGATTCATAGATCTCTTAGCTACCATTACCGTACTC
*RTG3* Forward	AGCTTGGGTGGTCATATGGCCATGGAGGCCATGATGAACAATAACGAAAGT
*RTG3* Reverse	GTTTTTCAGTATCTACGATTCATAGATCTCCTACCCCGAACCAAATTCTAA
*CST6* Forward	AGCTTGGGTGGTCATATGGCCATGGAGGCCATGTTTACTGGTCAGGAGTAT
*CST6* Reverse	GTTTTTCAGTATCTACGATTCATAGATCTCTTTATCTTTTCAGAATT
RT-PCR	
TSC22-1v1 Forward	AGGGAGAGCACTAGTGGGAGT
TSC22-1v1 Reverse	ATCTGTGACTGAGAAATACTC
TSC22-1v2 Forward	TTGGTTCAAAGTGTTAGTCAA
TSC22-1v2 Reverse	ATAGCTACCACACTTGCACCA
TSC22-1v3 Forward	TGGCTGCAATTGCATGAAATC
TSC22-1v3 Reverse	GCAATGAAATGGGTGACTGTG
β-actin Forward	GTGGGCCGCCCTAGGCACCAG
β-actin Reverse	CTCTTTGATGTCACGCACGATTTC

### Yeast growth and transformations

Yeast cells were routinely grown in synthetic minimal media containing Yeast nitrogen base (YNB), 2% glucose and the required amino acids or base. Glucose was replaced with 2% galactose and raffinose for experiments in which induction of the *GAL1* promoter was required. *ERG6* overexpression experiments performed with the KTY1/KTY3 strains required the use of YNB, 1% each of galactose and glucose to achieve wild-type growth rates as described in (Tedrick *et al.*, 2004). Transformations were performed using lithium acetate and selection of transformants was achieved by omitting specific amino acids or base for which auxotrophy was conferred by the vectors.

### Clonogenicity and cell survival assays

To assess the growth of yeast transformants expressing Bax under the control of a galactose inducible promoter, freshly saturated overnight cultures grown in selective glucose media were diluted 10-fold in water and normalized for cell number. 7.5-fold serial dilutions of these suspensions were spotted on galactose (inducing) and glucose (noninducing) media and thereafter incubated for 3–5 days at 30 or 37 °C as indicated. Results shown are representative of at least three independent experiments. For treatments with H_2_O_2_, saturated overnight cultures were diluted in fresh, galactose-containing media, incubated for 4 h to induce gene expression, and subsequently treated with the indicated concentration of H_2_O_2_. In some experiments, cells treated in this manner were plated directly onto minimal media and either irradiated with 100 J m^−2^ with UV light or incubated at 38 °C. Aliquots of different 7.5-fold serial dilutions were then spotted on selective, glucose-containing minimal media. For experiments involving yeast cells lacking *NKP1*, the outgrowth period in galactose-containing media was 6 h. Viability was determined using the vital dye trypan blue (Yang *et al.*, 2006). Samples were removed at the indicated time points, incubated for 5 min with 0.1% trypan blue and subsequently examined microscopically. Similar results were observed in at least three different experiments for all the growth assays shown.

### Reverse transcriptase (RT)-PCR

RNA was extracted from cultured cells and tissue samples using RNAzol as previously described (Jean-Baptiste *et al.*, 2005; Yang *et al.*, 2005). One μg of total RNA was reverse transcribed and amplified by PCR using the ThermoScript RT-PCR system (Invitrogen). Equal aliquots of cDNA were used to amplify the various TSC22-1 transcripts using the following conditions: 94 °C for 30 s, 57 °C for 30 s, 72 °C for 40 s for a total of 35 cycles. The primers used are described in Table 1. β-Actin mRNA was amplified as previously described (Jean-Baptiste *et al.*, 2005; Yang *et al.*, 2005). An aliquot of each PCR reaction was separated by electrophoresis on a 1.5% agarose gel stained with ethidium bromide, visualized and photographed under UV illumination.

### ROS detection

To detect accumulated intracellular ROS, aliquots of overnight cultures of yeast grown in selective glucose media were washed with sterile water, resuspended in galactose-containing media at an OD_600 nm_ of *c*. 0.1, and incubated at 30 °C. After 12 h, dihydrorhodamine 123 (DHR 123) was added to final concentration of 0.1 mg mL^−1^ and the cultures were further incubated for an additional 2 h. Cells were washed twice with water and visualized using fluorescent microscopy through a rhodamine optical filter (Zeiss Axiovert). The images were photographed and analyzed using northern eclipse software.

### Induction of YCA1-induced apoptosis

Plasmid pFM21 (a gift from F. Madeo) which contains a HA tagged *YCA1* gene expressed under control of the *GAL1* promoter was used to express *YCA1*. To assess the effects of TSC22^(86)^ expression on *YCA1* mediated apoptosis was assessed in BY4741 cells harbouring pFM21 as described (Madeo *et al.*, 2002). Briefly, exponentially growing cells in galactose minimal media were treated with 0.4 mM H_2_O_2_ for 16 h in order to activate yca1p. Under these conditions, the viability of control cells expressing TSC22^(86)^ alone was 94±4% while cells harbouring the *YCA1* expressing plasmid was decreased 58.66 (±4.48) %. Viability was determined using the vital dye trypan blue as described above.

### Genome-wide yeast two-hybrid screen

The pODB-TSC22^(86)^ construct was transformed into yeast strain pJ69-4α. Two individual clones resulting from this transformation were mated against the activation domain (Gal4p-AD) array in PJ69-4a, as described (Uetz *et al.*, 2000; Hazbun *et al.*, 2003). Diploids that grew in media lacking histidine, indicating expression of the *HIS3* gene under the promoter of the *GAL1* gene, were scored as putative interaction partners. Yeast strains expressing the Gal4p-AD–yeast ORF fusions corresponding to the eight positives identified in the genome-wide assay were selected from the array, and rescreened in a small-scale format against strains expressing Gal4p-DBD-TSC22. Strains expressing the Gal4p DNA-binding domain and activation domain, as well as a well-established interacting pair (Rad17p/Mec3p), were included as specificity controls.

### Yeast two-hybrid assay

Different combinations of the two-hybrid plasmids were transformed into the yeast strain DSY-1 (*MATa his3*Δ*200 trp1-901 leu2-3,112 ade2 LYS2∷(lexAop)*_*4*_*-HIS3 URA3∷(lexAop)8-lacZ GAL4*). Freshly saturated cultured of the transformed were serially diluted and spotted onto YNB glucose agar plates with and without histidine (Yang *et al.*, 2007).

## Results

### Bax suppressor 51 (Sup.51) represents the C-terminal region of Tsc22 and prevents the effects of apoptotic stimuli in yeast

**Fig. 1:**
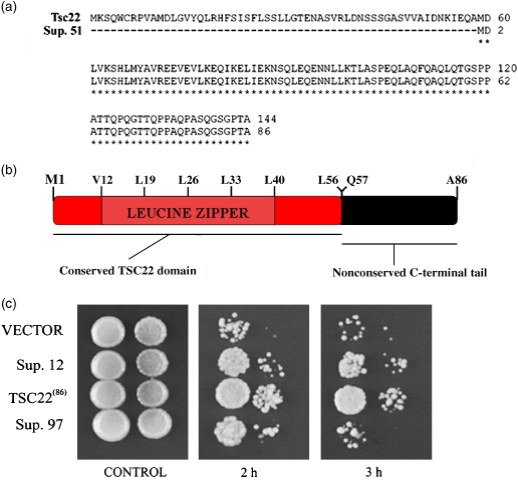
Sup. 51 represents the C-terminal region of Tsc22 and protects yeast cells from the effects of H_2_O_2_. (a) The amino acid sequence of Sup. 51 is shown aligned to the 144-residue human Tsc22 (GenBank accession #NT_024524). (b) A schematic representation of the domain organization of Tsc22^(86)^. ‘The conserved TSC-22 domain’ shown in red. The locations of the leucine (L) and valine (V) residues that form the LZ are shown. The domain spans residues Met^1^(M1)-Leu^56^(L56), and is succeeded by a variable C-terminal region spanning residues Gln^57^(Q57)-Ala^86^(A86). The predicted ‘LZ’ motif embedded within the TSC22 domain is present between residues V12-L40. (c) Wild Type yeast cells transformed with the indicated constructs were grown to logarithmic phase in galactose-containing minimal media. Aliquots of each culture were normalized for cell number, serially diluted, and then spotted on nutrient-containing agar media (CONTROL). Four (4) millimolar H_2_O_2_ was added to each culture and aliquots were removed after 2 and 3 h, serially diluted, and spotted on nutrient-containing media. All plates were incubated for 3 days at 30°C.

An 86 aa ORF corresponding to the C-terminus of the 144 aa human Tsc22 protein was previously identified as a suppressor of the lethal effects of expressing a proapoptotic Bax cDNA in yeast (Yang *et al.*, 2006). The N-terminal 56 residues of this clone represents the conserved TSC22 domain (Fig. 1b) (Kester *et al.*, 1999). Although Bax expression in yeast leads to apoptosis, it remains possible that the expression of a cDNA may suppress the effects of Bax by simply promoting cell growth. As a first step towards characterizing our TSC22 clone, henceforth called TSC22^(86)^, we determined if it could prevent the effects of other apoptosis-inducing stimuli when expressed in yeast. Elevations in the levels of intracellular ROS trigger apoptotic cell death in both yeast and mammalian cells (Zamzami *et al.*, 1995; Madeo *et al.*, 1999). A low dose of H_2_O_2_ has been shown to promote apoptotic cell death by increasing intracellular ROS and is commonly used to induce yeast apoptosis. Yeast cells expressing the TSC22^(86)^ clone displayed a markedly enhanced ability to form colonies on solid growth media after treatment with 4 mM H_2_O_2_, when compared to cells harboring empty vectors (Fig. 1c). This effect was observed at both 2 and 3 h posttreatment. These data indicate that Tsc22^(86)^ likely functions as an antiapoptotic protein in yeast (see also below).

### The human *TSC22* gene encodes for multiple transcripts that specify different proteins with prosurvival functions in yeast

**Fig. 2:**
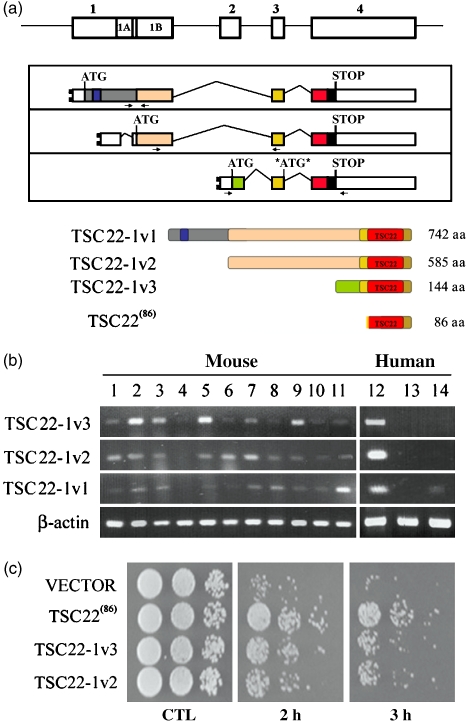
Analysis and characterization of the alternatively spliced TSC22 transcripts. (a) A schematic representation of the genomic organization of human *TSC22*. The sequences of all human TSC22 cDNAs available in GenBank were compared to the sequence of the human genome using blast in order to determine the exon composition of the *TSC22* gene. The four exons of *TSC22* are shown at the top of the Figure as boxes numbered ‘1’–‘4’. The locations of the cryptic exons ‘1a’ and ‘1b’ that are present within exon 1 are indicated. The exon composition of the different TSC22 transcripts is shown boxed. The locations of the translational start (ATG) and stop (STOP) codons are also indicated. The coding sequences are shown in color while the 5′ and 3′ UTRs are shown as empty boxes. Arrows are used to depict the location of the oligos used for RT-PCR. At the bottom are representations of the three predicted Tsc22 proteins produced by the alternatively spliced transcripts. Different regions of the proteins are color-coded and correspond to the similarly colored mRNA sequence from which they are encoded. The region of the conserved TSC22 box is shown in red. The names of the different proteins are shown at the left (‘v’= splice variant), while their sizes are indicated at the right. The 86 aa protein encoded by the TSC22^(86)^ cDNA is also shown. The location of the start codon (ATG) for this cDNA is shown as ‘^*^ATG^*^’. (b) RT-PCR analysis of the expression of the three TSC22 transcripts. Total RNA was isolated from a variety of mouse tissues and cell lines (MOUSE), as well as human cells (HUMAN). RT-PCR was used to amplify the TSC22 transcripts using specific oligos. The different mouse tissues are: 1, brain; 2, heart; 3, lung; 4, liver; 5, spleen; 6, kidney; 7, testis; 8, skeletal muscle; 9, HL-1; 10, C2C12+vehicle; 11, C2C12+TNF-α (50 ng mL^−1^). The human cell lines are: 12, HEK293; 13, fetal brain; 14, skeletal muscle cDNA libraries. β-Actin was also amplified using specific oligos and served as an internal control. (c) Wild Type yeast cells transformed with vectors expressing the indicated Tsc22 proteins were grown to logarithmic phase in galactose-containing minimal media. Aliquots of each culture were normalized for cell number, serially diluted, and then spotted on nutrient-containing agar media before (CTL) and 2 or 3 h after treatment with 4 mM H_2_O_2_. The plates were incubated for 3 days at 30°C.

In addition to the previously characterized 144-residue Tsc22 protein, the database searches of human proteins using Tsc22^(86)^ revealed identical matches with the C-terminal 86 aa of two other Tsc22 proteins of 585 and 742 aa. The three differently sized Tsc22 proteins share an identical 86 residue C-terminal region but differ in their N-terminal portions (Fig. 2a). The common C-terminus is comprised partly by 56 residues that are defined as a TSC22 domain (Kester *et al.*, 1999). The remaining portion that is identical between the three Tsc22 proteins is comprised of the 30 residues that serve as the C-terminal tail. These observations suggested that the *TSC22* gene is likely alternatively spliced to produce multiple transcripts. In spite of this diversity, the 144-residue isoform is the one that is commonly referred to as Tsc22 (Kawamata *et al.*, 2004).

In order to investigate the origin of the different TSC22 transcripts and proteins, we set out to characterize the organization of the human *TSC22* gene. To accomplish this, we used blast to compare the sequences of the different TSC22 cDNAs to the sequence of the human genome. The *TSC22* gene was found to consist of four different exons that are spread over at least 141.6 kb of DNA found on chromosome 13 (Fig. 2a). The common 56 aa containing the TSC22 domain and C-terminal 30 residues, as well as the translational stop site are all encoded by exon 4 exclusively (Fig. 2a). The 18-aa sequence at the N-terminus of the TSC22 domain that is common to all three proteins is encoded by exon 3. All three TSC22 transcripts contain exons 3 and 4. The observed differences between the three TSC22 transcripts are due to alternative splicing of exons 1 and 2 (Fig. 2a). The alternatively spliced variant 3 (TSC22v3) contains exon 2 at its 5′ end (Fig. 2a). This exon contains the predicted translational start site and the unique N-terminus that is present only in the 144 aa Tsc22v3 protein (Fig. 2a). The TSC22v1 mRNA consists of exons 1, 3 and 4 (Fig. 2a). Exon 1 provides its 5′ end, the predicted translational start site and the unique N-terminus that is present only in the 742-residue Tsc22v1 protein (Fig. 2a). In contrast, the TSC22v2-encoded 585-residue protein is an N-terminally truncated form of the 742-residue TSC22v3 protein (Fig. 2a). The 5′ end of the TSC22v2 transcript consists of exons 1A and 1B (Fig. 2a). These are cryptic exons that are part of the larger exon 1 used to make the 5′ end of the TSC22v1 transcript. The translational start site used to make the 585-residue TSC22v2 protein is predicted to be at the 5′ end of exon 1B (Fig. 2a).

The sizes of the different exons and the sequences of the intron/exon boundaries of the *TSC22* gene were determined by comparing the sequences of the genomic DNA and the sequences of the different cDNAs (Table 2). All the exon/intron boundaries follow the GT/AG rule for splice sites.

**Table 2:** Sequences of the exon–intron junctions of the alternatively spliced human *TSC22-1* gene

Exon*	Exon size (bp)	Splice acceptor site (intron–EXON)	Splice donor site (EXON–intron)
1	>1957	5′end	GAGAG-gtaag
1a	>140	5′end	ATAAA-gtggt
1b	1456	ggaag-TGGAG	GAGAG-gtaag
2	307	5′end	AATAG-gtaaa
3	52	ttcag-CTCCT	CTATG-gtatg
4	1362	tccag-GATCT	TTGAT=polyA

The intron/exon boundaries were determined by comparing the sequences of the longest human cDNAs corresponding to the three different TSC22-1 transcripts (GenBank accession numbers: AK027071 for TSC22-1v1; CR627459 for TSC22-1v2; U35048 for TSC22-1-v3) with the corresponding genomic DNA sequence found on chromosome 3 (GenBank accession number NT_024524). Introns are shown in lower case and exons in upper case letters.

* Exons 1, 1a, 2 are used for the 5′ ends of TSC22-1v1, v2, and v3 transcripts, respectively. Exons 1a and 1b contain alternately spliced regions of exon1 and are both used to encode the TSC22-1v1 transcript.

To confirm that the cDNAs are not artifacts of the cDNA library from which they were cloned and actually represent endogenously expressed transcripts, we performed an RT-PCR analysis of the *TSC22* gene products using primers that recognized both the human- and mouse-encoded transcripts (Fig. 2b). Three sets of primers were used in order to specifically detect the transcripts. We observed expression of TSC22v3 in a number of mouse tissues with prominent levels of the transcript detected in the heart, lung, spleen, and the cardiac HL-1 cell line (Fig. 2b). The TSC22 v1 transcript was also detected in a range of mouse tissues. Due to the lack of unique regions in the v2 transcript with respect to v1, we were unable to ascertain the expression pattern for v2 alone. Nevertheless, we found that v2 is expressed in brain, spleen, kidney, and testis since v1 was not detected in these tissues. We also examined the expression of TSC22-encoded transcripts in human cells. We found readily detectable levels of the v1 and v3 transcripts in HEK293 cells, but were unable to detect expression in cDNA libraries prepared from either human fetal brain or skeletal muscle extracts (Fig. 2b). β-Actin was detected in all tissues and cells examined and served as a loading control for these studies. This analysis indicates that three *TSC22* gene products are expressed endogenously in a variety of mouse tissues and at least two of these transcripts are expressed in a human cell line.

The TSC22 cDNA that we isolated in our Bax suppressor screen, TSC22^(86)^, encodes for the 86 C-terminal residues of Tsc22 that is present in all three Tsc22 proteins produced by the alternately spliced *TSC22* gene (Fig. 2a). It consists of the entire 4th exon and also contains the 3′ end of exon 3 that supplies its predicted translational start site (Fig. 2a). Given that there is no unique sequence present in the TSC22^(86)^ cDNA, we were unable to use RT-PCR to determine if it represents a fourth TSC22 splice variant or if the cDNA represents a truncated version of one of the 3 known TSC22 transcripts. We therefore wondered if the antiapoptotic effect observed with the expression of TSC22^(86)^ is a potential artifact due to the expression of a truncated form of TSC22. We thus sought to determine whether the endogenously expressed TSC-22 gene products could also function to promote cell survival in yeast. Cells transformed with vectors expressing a cDNA encoding the 144-residue Tsc22v3 displayed an enhanced ability to grow, as compared to empty vector-transformed cells, after treatment with H_2_O_2_ (Fig. 2c). This effect, noted after both a 2- and a 3-h treatment, was also observed with cells transformed with a vector expressing a cDNA encoding the 585-residue Tsc22v2 (Fig. 2c). The prosurvival effect was consistently less marked than that conferred by TSC22^(86)^. While we have not compared expression levels, this could explain the differences in the protective effect of the different Tsc22 isoforms. Nevertheless, these observations demonstrate that Tsc22 proteins have a pro-survival effect in yeast.

### The conserved TSC22 domain defines a four-member gene family

**Fig. 3:**
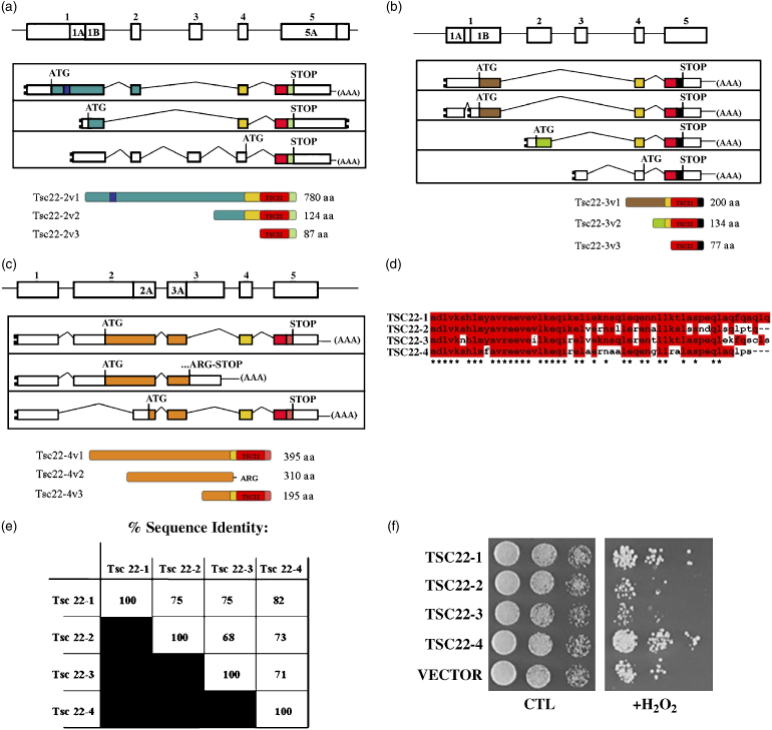
TSC22 is a member of a multi-gene family with other antiapoptotic members. Schematic representations of the organization of human *TSC22-2* (a), *TSC22-3* (b), and *TSC22-4* (c) genes. The sequences of all human TSC22-related cDNAs available in GenBank were compared to the sequence of the human genome using blast in order to determine the exon composition of the TSC22 genes. The five exons of each gene are shown at the top of the Figure as boxes numbered ‘1’–‘5’. The locations of the cryptic exons ‘1a’ and ‘1b’ that are located within exon 1 of *TSC22-2* and *TSC22-3* are indicated. Similarly, Cryptic exon ‘5a’ is indicated in exon 5 of *TSC22-2*, as well as cryptic exons ‘2a’ and ‘3a’ in exons 2 and 3, respectively of *TSC22-4*. The exon composition of the different TSC22 transcripts is shown boxed. The locations of the translational start (ATG) and stop (STOP) codons are also indicated. The coding sequences are shown in color while the 5′ and 3′ UTRs are shown as empty boxes. At the bottom are representations of the three predicted Tsc22 proteins produced by the alternatively spliced transcripts. Different regions of the proteins are color-coded and correspond to the similarly colored cDNA sequence from which they are encoded. The region of the conserved TSC22 box is shown in red. The names of the different proteins are shown at the left (‘v’= splice variant), while their sizes are indicated at the right. (d) An alignment of the TSC22 domains of the four Tsc22 family members. Residues identical to Tsc22-1 are shown in red. An asterisk (^*^) is used to indicate residues that are identical between all four sequences. (e) The amino acid sequences of the different TSC22 sequences from all four human genes were compared in a pair-wise manner. The percentage of sequence identity between the different family members was determined and is shown boxed. (f) Wild-type yeast cells transformed with the indicated vectors were grown to logarithmic phase in galactose-containing minimal media. Aliquots of each culture were normalized for cell number, serially diluted, and then spotted on nutrient-containing agar media before (CTL) and 2 or 3 h after treatment with 4 mM H_2_O_2_. The plates were incubated for 3 days at 30°C.

Analysis of the *TSC22* sequence in GenBank also revealed a large number of human cDNAs that contained regions with high sequence identity to TSC22^(86)^. The highest sequence identity was present in the regions coding for the TSC22 domain. This suggested that the conserved TSC22 domain is part of a multi-gene family. Although most published studies pertaining to Tsc22 refer to the 144-residue product of the TSC22v3 transcript we described in the previous section (Fig. 2a), a level of ambiguity exists in the current literature as to which TSC22 containing gene and which alternatively spliced variant is actually being studied. In order to clearly describe the human gene family and classify the various gene products, we used blast to compare the sequences of all the available human cDNAs to the sequence of the human genome. The results of this analysis revealed that there are four different human genes containing TSC22 domain-encoding regions (Figs 2 and 3). For clarity and to conform to the information available in some of the published reports, we named the genes *TSC22-1* to *TSC22-4* (Fiol *et al.*, 2007). *TSC22-1* corresponds to the founding member of the gene family and to the *TSC22* gene described in Fig. 2 (Jay *et al.*, 1996). Human *TSC22-2* consists of five exons that are spread over at least 51.1 kb of DNA located on chromosome 3 (Fig. 3a). Three different transcripts (v1 to v3) that are produced by alternative splicing were identified from the *TSC22-2* gene. Three different proteins all containing the TSC22 domain, ranging in size from 84 to 710 aa are also predicted to be produced (Fig. 3a). Human *TSC22-3*, which encodes the well-studied GILZ protein (Asselin-Labatt *et al.*, 2004), also consists of five exons that are spread over at least 62.6 kb of DNA that is located on chromosome X (Fig. 3b). Three different transcripts (v1–v3) that are produced by alternative splicing were identified from the *TSC22-3* gene. Three different proteins all containing the TSC22 domain, ranging in size from 77 to 200 aa are also predicted to be produced (Fig. 3b). Human *TSC22-4* also consists of five exons that are spread over at least 12.7 kb of DNA that is located on chromosome 7 (Fig. 3c). Similar to the other *TSC22* genes, *TSC22-4* is also predicted to generate three different transcripts (v1–v3) that are produced by alternative splicing. Three different proteins ranging in size from 195 to 395 aa are also predicted to be produced (Fig. 3c). In contrast to the other three *TSC22* genes, only two of the *TSC22-4* proteins, Tsc22-4v1 and Tsc22-4v3, contain the TSC22 domain. TSC22-4v2 is made up of exons 1–3 and lacks exons 4 and 5 that encode for the TSC22 domain. It should be noted that the *TSC22-2* and *TSC22-3* genes produce a respective 87 and 77 residue protein that contains the TSC22 domain at their N-terminus, similar to the 86 aa protein encoded by our TSC22^(86)^ clone. This indicates that the *TSC22-1* gene encoding an 86-residue splice variant would not be unique in this regard and suggests that the TSC22^(86)^ may represent a *bona fide* TSC22-1 transcript.

The sizes of the different exons and the sequences of the intron/exon boundaries were determined for TSC22-2, -3 and -4 by comparing the sequences of the genomic DNA and the sequences of the different cDNAs (Tables 3–5). All the exon/intron boundaries follow the GT/AG rule for splice sites.

**Table 3:** Sequences of the exon–intron junctions of the alternatively spliced human *TSC-22-2* gene

Exon*	Exon size (bp)	Splice acceptor site (intron–EXON)	Splice donor site (EXON–intron)
1	>2308	5′end	GACAG-gtatg
1a	>130	5′end	GACAG-gtatg
1b	>101	5′end	GACAG-gtatg
2	72	cacag-GAATC	GATAG-gtatg
3	157	gctag-AATTT	AATTG-gtaag
4	52	tccag-TGCAT	CAATG-gtaag
5	1469	cacag-GATCT	3′end
5a	>1742	cacag-GATCT	TTTCT=3′end-polyA

The intron/exon boundaries were determined by comparing the sequences of the longest human cDNAs corresponding to the three different TSC-22-2 transcripts (GenBank accession numbers: NM_014779 for TSC-22-2-V1; AF201291 for TSC-22-2-V2; AF201292 for TSC-22-2-V3) with the corresponding genomic DNA sequence found on chromosome 2 (GenBank accession number NT_005612). Introns are shown in lower case and exons in upper case letters.

* Exons 1, 1a, and 1b are used for the 5′ ends of TSC-22-3-V1, V2, and V3 transcripts, respectively. Exons 1a and 1b contain alternate 3′ portions of exon1 and are used for two different transcripts encoding TSC-22-2-V3 and TSC-22-2-V2, respectively. Exon 5a contains an alternately spliced region of both TSC-22-2-V1 and TSC-22-2-V3 transcripts.

**Table 5:** Sequences of the exon–intron junctions of the alternatively spliced human *TSC22-4* gene

Exon*	Exon size (bp)	Splice acceptor site (intron–EXON)	Donor acceptor site (EXON–intron)
1	>486	5′end	GGCCCCAGAG-gtagggttca
2	1031	ctttttccag-TTTGCAAACT	ATGGGGGCAG-gtaagacctg
2a	183	accccagcag-CGCCCCCCAG	ATGGGGGCAG-gtaagacctg
3	543	ccgcctccag-GTGCCCCCAC	GAAACTTGAA=3′end-polyA
3a		ccgcctccag-GTGCCCCCAC	ACGATGATAG-gtaggtgggc
4	49	gtctctccag-TGGCTCCGGA	GCAAGCCATG-gtaagagaag
5	650	gggcccccag-GACTTGGTGA	GAAACTTGAA=3′end-polyA

The intron/exon boundaries were determined by comparing the sequences of the longest human cDNAs corresponding to the three different TSC22-4 transcripts (GenBank accession numbers: BC001486 for TSC22-4v1; BC031622 for TSC22-4v2; CR606606 for TSC22-4v3) with the corresponding genomic DNA sequence found on chromosome 7 (GenBank accession number CH236956). Introns are shown in lower case and exons in upper case letters.

* Exon 1 is present at the 5′ end of all four TSC-22 #4 transcripts. Exon 2a contains a 3′ portion of exon2 and is used for the TSC22-4v2 transcript. Exon 3a contains the 5′ portion of exon 3 and is used for the TSC22-4v1 and v3 transcripts.

**Table 4:** Sequences of the exon–intron junctions of the alternatively spliced human *TSC-22-3* gene

Exon*	Exon size (bp)	Splice acceptor site (intron–EXON)	Splice donor site (EXON–intron)
1	>688	5′end	CACAG-gtggg
1a	>162	5′end	CACAG-gtccg
1b	412	tccag-GCACC	CACAG-gtggg
2	>372	5′end	AACAG-gtaac
3	>167	5′end	CGCCG-gtagg
4	52	ggcag-TGCCT	CCATG-gtgag
5	1529	ggcag-GATCT	TTTCT=3′end-polyA

The intron/exon boundaries were determined by comparing the sequences of the longest human cDNAs corresponding to the four different TSC-22 #3 transcripts (GenBank accession numbers: NM_198057 and AK092645 for TSC-22-3-V1; NM_004089 for TSC-22-3-V2; NM_00101588 for TSC-22-3-V3) with the corresponding genomic DNA sequence found on chromosome X (GenBank accession number NT_011651). Introns are shown in lower case and exons in upper case letters

* Exons 1, 2, and 3, are used for the 5′ ends of TSC-22-3-V1, V2, and V3 transcripts, respectively. Exons 1a and 1b contain respective 5′ and 3′ portions of exon1 and are used for two different transcripts specifying a unique protein, TSC-22-3-V1.

The Tsc22-1v1 and Tsc22-2v1 proteins have similar sizes (742 and 780 aa, respectively) and they display a 30% identity throughout this sequence, suggesting a common ancestry (not shown). Apart from this, the sequence similarity between the four Tsc22 proteins is largely limited to the TSC22 domain. The amino acid sequences of the TSC22 domain of all four proteins are shown aligned in Fig. 3d. All four proteins display a number of conserved residues including the presence of the previously described putative LZ motif (Jay *et al.*, 1996). Pair-wise comparisons revealed that the TSC22 domain of Tsc22-1 is most similar to that of Tsc22-4 (84% sequence identity) while the TSC22 domains of Tsc22-2 and Tsc22-3 display slightly lower sequence identity at 75% each (Fig. 3e). Although the TSC22 domain is highly conserved across a number of metazoan species, its precise function is at present uncertain. With the exception of the predicted LZmotif, there are no other proteins with similar sequences in the GenBank database. Similarly, we found no clues as to the function of the non-TSC22 portion of Tsc22^(86)^ in GenBank. We did discover a 46 aa region within the N-terminal non-TSC22 regions of the longest Tsc22 proteins, Tsc22-1v1 and Tsc22-2v1, that display considerable sequence identity (50% identity between residues 59–103 of Tsc22-1v1 aligned to residues 145–190 of Tsc22-2v1) (Figs 2 and 3). These sequences are not present in other proteins in GenBank and their functional significance is at present unknown.

The 144-residue product of the *TSC22-1* gene has been shown to be either pro- or antiapoptotic depending on the cell type examined (Kawamata *et al.*, 2004). Although not all TSC22 domain-containing proteins have been tested, it is probable, based on sequence similarity, that other members of this family are also involved in mediating or preventing apoptosis (Asselin-Labatt *et al.*, 2004). Because the products of the *TSC22-1* gene can protect from the effects of apoptotic stimuli in yeast (Fig. 2), we examined the potential pro-survival functions of all Tsc22 proteins in yeast by expressing cDNAs corresponding to the TSC22 domains (including the variable C-terminal tail) from the four *TSC22* genes. Yeast transformants expressing the individual TSC22 domains were challenged with H_2_O_2_, diluted and spotted onto nutrient agar plates to determine their ability to grow. In addition to that from *TSC22-1*, the TSC22 domain of *TSC22-4* was also found to confer resistance to H_2_O_2_ (Fig. 3f). This indicates that the anti-apoptotic property of TSC22 is not limited to the motif encoded by *TSC22-1*, but extends to that encoded by *TSC22-4* as well.

### TSC22^(86)^ is antiapoptotic in yeast

**Fig. 4:**
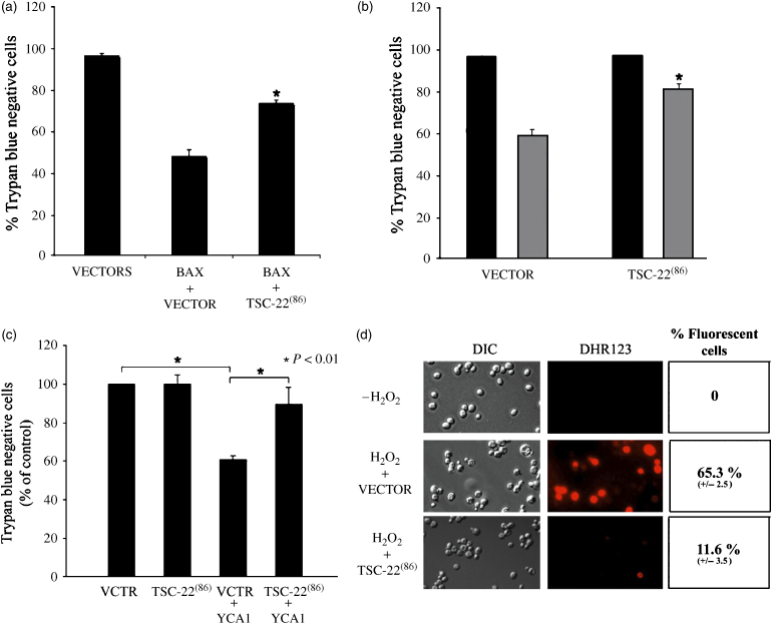
Overexpression of human TSC22^(86)^ is antiapoptotic in yeast. (a) Wild-type cells harbouring an empty plasmids (VECTORS), a plasmid expressing Bax alone (BAX) or in combination with a plasmid expressing TSC22^(86)^ were grown to saturation, diluted in fresh galactose-containing media, and incubated for 16 h at 30°C. Viability was determined from aliquots of cells stained with the vital dye trypan blue by visualization with light microscopy. Data are expressed as the percentage of cells that do not stain blue with the dye (% Trypan blue negative) and represent the mean of three independent experiments (±SEM) ^*^, Student's *t*-test *P*<0.01. (b) Wild-type cells transformed with either an empty vector (VECTOR) or a TSC22^(86)^-expressing plasmid were grown to logarithmic phase and treated with 5 mM H_2_O_2_ (grey bars) or left untreated (black bars). Viability was determined using the vital dye trypan blue as in Fig. 4a. Data represent the mean of three independent experiments (±SEM). ^*^, Student's *t*-test *P*<0.01. (c) Wild-type cells harboring the indicated combinations of empty vectors (VCRT) or *YCA1*- and TSC22^(86)^-expressing plasmids were incubated with 0.6 mM H_2_O_2_ for 16 h. Viability was determined using with the vital dye trypan blue as in Fig. 4a. Data represent the mean of three different experiments (±SEM; Student's *t*-test: *P*<0.01 (d) Yeast cells harboring an empty vector (VECTOR) or the TSC22^(86)^ expressing plasmid were grown in galactose-containing media to early logarithmic phase then incubated with or without 4 mM H_2_O_2_ for 2 h. The cultures were further incubated for 2 h with 0.1 mg mL^−1^ of DHR 123 for a further 2 h. Samples of the different cultures were removed and examined by light (DIC) and fluorescent light (DHR 123) microscopy. Representative photographs are shown and the calculated proportion of 300 cells displaying a fluorescent signal (±SEM) is indicated in boxes at the right.

An antiapoptotic protein should, by definition, prevent cell death in response to apoptotic stimuli (Kroemer *et al.*, 2005). To examine if TSC22^(86)^ is antiapoptotic in yeast, we directly assayed for the viability of TSC22^(86)^-expressing cells following apoptotic stimuli. Microscopic examination of cells stained with the vital dye trypan blue revealed that 47.8 (±3.0) % of cells excluded the dye after 16 h of induction of the expression of mouse BAX (Fig. 4a). In contrast, 73.2 (±1.4) % of cells coexpressing TSC22^(86)^ retained viability, indicating the ability of TSC22^(86)^ to protect from the lethal effects of BAX expression (Fig. 4a). Similarly, 59.7 (±3.0) % of empty vector-transformed cells remained viable after a 5 h treatment with 5 mM H_2_O_2_, while the proportion of viable cells was significantly higher at 82.0 (±2.6) % in yeast expressing TSC-22^(86)^ (Fig. 4b).

Yeast cells produce a caspase-like protein encoded by the *YCA1* gene that serves to induce apoptosis (Madeo *et al.*, 2002). Evidence for the importance of *YCA1* comes from studies showing that its overexpression induces apoptosis, while yeast cells lacking this gene display increased resistance to apoptosis-inducing stresses. We therefore tested the effect of TSC22^(86)^ on the cell death induced by *YCA1* overexpression (Fig. 4c). The viability of yeast cells harbouring different combinations of plasmids was determined using a vital dye. The viability of cells overexpressing *YCA1* is decreased to 58.7 (±4.5) % when compared to control cells harbouring an empty vector. The coexpression of TSC22^(86)^ with *YCA1* resulted in an increased viability of cells to 92.9 (±7.3) % (Fig. 4c).

Finally, it is well established that many apoptotic stimuli, including H_2_O_2_, induce cell death in both mammalian and yeast cells by increasing the intracellular levels of ROS (Zamzami *et al.*, 1995; Madeo *et al.*, 1999). An increased production of endogenous ROS is known to occur during the apoptotic programme in yeast and is a commonly used marker for apoptosis (Frohlich *et al.*, 2007). We tested the effect of TSC22^(86)^ on the H_2_O_2_-induced accumulation of ROS detected with the cell-permeable probe DHR123, a molecule that is converted into the fluorescent compound rhodamine upon oxidation (Fig. 4d). Empty vector-transformed cells displayed an undetectable level of fluorescence, indicating the relatively low basal level of endogenous ROS present in these cells. After a 4-h treatment with H_2_O_2_ the proportion of fluorescent cells observed was 65.3 (±2.6) %. The percentage of fluorescent TSC22^(86)^-expressing cells was reduced to 11.6 (±3.5) %, suggesting that TSC22^(86)^ expression prevents the accumulation of endogenous ROS in yeast cells challenged with an apoptotic stimulus. Taken together, these data indicate an antiapoptotic function for TSC22^(86)^ in yeast.

### A genome-wide two-hybrid screen for TSC22^(86)^-interacting yeast proteins

**Fig. 5:**
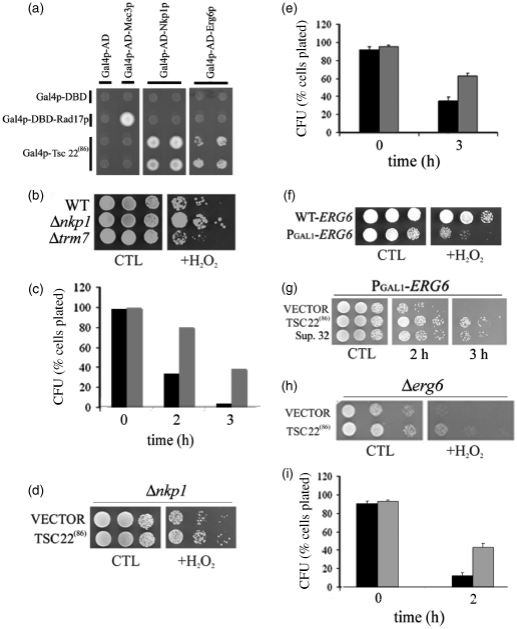
A genome-wide two-hybrid screen for TSC22^(86)^-interacting proteins. (a) Tsc22^(86)^ interacts specifically with Nkp1p and Erg6p by a yeast two-hybrid assay. Strains expressing Gal4 DNA-binding domain (Gal4DBD) or Gal4DBD fused to Rad17 or Tsc22^(86)^ were mated with yeast strains of the opposite mating type expressing Gal4p activation domain (Gal4AD) or Gal4AD fused to Mec3p, Nkp1p or Erg6p. Diploids were selected and pinned on media lacking tryptophan, leucine and histidine, and containing 1.5 mM 3-aminotriazole. The Rad17-Mec3 pair serves as a positive control in this assay. (b) Wild-type (WT), Δ*nkp1*, and Δ*trm7* cells were incubated in minimal media to logarithmic phase before treatment with 4 mM H_2_O_2_ for 2 h. Samples of each culture were removed, normalized for cell number, and spotted on nutrient-containing agar media and incubated for 2–3 days. (c) WT (black bars) and Δ*nkp1* mutant (grey bars) cells were incubated in minimal media to logarithmic phase. Aliquots of each sample were removed prior to, as well as after 2 and 3 h of, treatment with 4 mM H_2_O_2_. At each time point, an equal number of cells (250) from each sample were plated on nutrient-containing agar media. The number of colonies formed was counted after 2 days of incubation at 30°C. The data are expressed as the percentage (%) cells that were plated that are capable of forming colonies (CFU). The data are representative of two different experiments. (d) Δ*nkp1* mutant cells transformed with either an empty vector (VECTOR) or a TSC22^(86)^-encoding vector were grown to logarithmic phase in galactose-containing minimal media. Separate cultures of each sample were left untreated (CTL) or treated with 4 mM H_2_O_2_ (+H_2_O_2_). After 3 h of further incubation, aliquots were removed and normalized for cell number, serially diluted, then spotted on nutrient-containing agar media and incubated for 2–3 days. Photographs of the plates are shown. (e) Δ*nkp1* mutant cells transformed with either an empty vector (black bars) or a TSC22^(86)^-encoding plasmid (grey bars) were grown to logarithmic phase and treated with 4 mM H_2_O_2_. Aliquots were removed at the times indicated, and 250 cells from each sample were plated on nutrient-containing agar media. The number of colonies formed was counted after 2 days of incubation at 30°C. The data re expressed as the percentage (%) cells that were plated that are capable of forming colonies (CFU). Data represent the mean of three independent experiments (±SEM). (f) Galactose-inducible *ERG6*-overexpressing strain KTY3 (P_GAL1_-*ERG6*) and parental wild-type strain KTY1 (WT-*ERG6*) were grown to early logarithmic phase in media containing glucose, galactose and raffinose each to 1%. Cells were either left untreated (CTL) or treated with H_2_O_2_ to 1.2 mM (+H_2_O_2_) for 2 h before spotting different serial dilutions of aliquots normalized for cell number on nutrient-containing agar media. Photographs of the plates after 3 days of incubation at 30°C are shown. (g) Cells of the galactose-inducible *ERG6*-overexpressing strain KTY3 (P_GAL1_-*ERG6*) transformed individually with either empty vector (VECTOR) or with the vectors expressing the indicated proteins were grown to early logarithmic phase in media containing glucose, galactose and raffinose each to 1%. Cells were either left untreated (CTL) or treated with 1.2 mM H_2_O_2_ (+H_2_O_2_) for 2 h before spotting different serial dilutions of aliquots normalized for cell number on nutrient-containing agar media. Photographs of the plates after 3 days of incubation at 30°C are shown. (H) Δ*nkp1* mutant cells transformed with either an empty vector (VECTOR) or a TSC22^(86)^-encoding vector were grown to early logarithmic phase in galactose-containing minimal media. Separate cultures of each sample were left untreated (CTL) or treated with H_2_O_2_ to 4 mM (+H_2_O_2_). After 2 h, aliquots were removed and normalized for cell number, serially diluted, then spotted on nutrient-containing agar media. Photographs of the plates after 3 days of incubation at 30°C are shown. (i) Δ*erg6* cells transformed with either an empty vector (black bars) or a TSC22^(86)^-encoding plasmid (grey bars) were grown to early logarithmic phase in treated with 4 mM H_2_O_2_. Aliquots were removed at the times indicated, and an equal number of cells (250) from each sample were spotted on nutrient-containing media. The number of colonies formed was counted after 2 days of incubation at 30°C. The data are expressed as the percentage (%) cells that were plated that are capable of forming colonies (CFU). Data represent the mean of three independent experiments (±SEM).

The yeast genome encodes 184 putative LZ-containing proteins, 19 of which are TFs [Comprehensive Yeast Genome Database, MIPS, (http://mips.gsf.de) (Mewes *et al.*, 2006)]. None of these proteins appear to be orthologs of Tsc22^(86)^. Despite this, we hypothesized that the pro-survival effect of expressing TSC22^(86)^ in yeast is the result of the protein competing with an endogenous LZ-containing TF protein for the formation of a dimeric complex. This hypothesis is based on the observation that Tsc22 appears to act as a transcriptional modulator in mammalian cells despite the absence of a transcriptionally active domain (Kester *et al.*, 1999). Further, TSC22 dimerization with an LZ-containing TF has also been proposed to be responsible for at least some of the functions of different Tsc22 proteins when overexpressed in mammalian cells (Kester *et al.*, 1999; Hino *et al.*, 2002). Nevertheless, only the Tsc22-4v3 protein has been identified as a Tsc22-1v3 binding partner, and this protein does not have an apparent yeast ortholog (Kester *et al.*, 1999). The yeast two-hybrid assay has been previously used to screen for interactions between LZ-containing proteins (Strathmann *et al.*, 2001; Weltmeier *et al.*, 2006). To try and identify potential Tsc22^(86)^-interacting proteins, we performed a genome-wide two-hybrid screen. Yeast strains carrying the Tsc22^(86)^ protein as a Gal4p DNA-binding domain hybrid were tested in duplicate against an array of *c*. 6000 yeast colonies. Each colony is derived from a transformation with a plasmid containing a DNA fragment encoding a full-length *S. cerevisiae* ORF fused to the Gal4p activation domain (Uetz *et al.*, 2000; Hazbun *et al.*, 2003). Eight positives resulting from this high-throughput screen were retested for their ability to interact with Tsc22^(86)^, resulting in the confirmation of two interactions: Nkp1p, which was originally identified as positive in both screens and that yielded strong growth on two-hybrid selective plates, and Erg6p, a positive that came up in a single screen and showed weaker growth on the selective media. Both interactions were specific, as shown by the inability of either the Nkp1p or Erg6p protein to interact with Gal4p-DNA-binding domain alone or a Gal4p-DNA-binding domain fused to Rad17p (Fig. 5a). Surprisingly, none of the 184 yeast LZ-containing proteins were identified as binding partners of Tsc22^(86)^. This is in spite of the fact that the array contains transformants for all of them, although it is not known whether the Gal4p activation domain fusion proteins are expressed.

Even though both Nkp1p and Erg6p lack any apparent LZ motif in their primary sequence, we examined the possibility that an interaction with either of these proteins was necessary for the antiapoptotic function of Tsc22^(86)^. We tested two possible hypotheses under which these interactions might be required. The first is that the Tsc22^(86)^ -interacting proteins are proapoptotic and Tsc22^(86)^ serves to inhibit this effect. A second scenario involves the formation of an antiapoptotic complex consisting of Tsc22^(86)^ and Nkp1p or Erg6p. We initially asked if Nkp1p has a role in yeast PCD responses. WT yeast cells treated with H_2_O_2_ for 2.5 h before plating on nutrient containing agar media display a reduced ability to form colonies when compared to untreated cells (Fig. 5b). As compared to WT, an isogenic strain deleted for *NKP1* displayed a marked resistance to the effects of H_2_O_2_. As a control we also examined yeast cells lacking the Nkp1p-interacting protein encoded by the *TRM7* gene. In contrast to Δ*nkp1* cells, Δ*trm7* cells displayed a hypersensitive response to H_2_O_2_ (Fig. 5b). To confirm the resistance observed in cells lacking NKP1, we assessed their colony forming ability when challenged with H_2_O_2_ after 2 and 3 h, respectively, 78.3% and 37.8% of cells remained clonogenic, while only 32.9% and 3.6% of WT cells formed colonies after these respective times of treatment (Fig. 5c). We next tested if TSC22^(86)^ retained its anti-apoptotic effect in yeast lacking *NKP1*. Transformants expressing TSC22^(86)^ displayed a marked resistance to the effects of H_2_O_2_ on clonogenicity of yeast (Fig. 5d and e). The proportion of TSC22^(86)^-expressing cells able to form colonies after 3 h of treatment was observed to be higher than that of vector-transformed cells (TSC22^(86)^, 62.9 (±2.9) %; VECTOR, 35.0 (±3.9) %; Fig. 5e). Thus despite the initial observation suggesting that *NKP1* is involved in apoptotic responses, we find that it is not required for the antiapoptotic effects of TSC22^(86)^.

In order to test the effect of Erg6p overproduction on PCD responses, we used a strain of yeast with the *GAL1* promoter inserted upstream of the *ERG6* gene (Tedrick *et al.*, 2004). It had been previously reported that induction of the *ERG6* gene (using 1% glucose and 1% galactose) was sufficient to promote increases in ergosterol levels (Tedrick *et al.*, 2004). Cells harbouring this insertion treated for 2.5 h with H_2_O_2_ displayed a marked reduction in growth as compared with the parental strain (Fig. 5f). This reduction in growth was significantly more pronounced than the modest reduction observed in untreated cells. These data suggest that increased sterol levels render cells hypersensitive to the effects of H_2_O_2_. We exploited this phenotype to ask whether TSC22^(86)^ promotes cell survival through a mechanism involving Erg6p. *ERG6*-overexpressing cells that are transformed with a TSC22^(86)^-expressing vector were more resistant to the effects of *ERG6* overexpression on clonogenicity, when compared to the same cells transformed with an empty vector (Fig. 5g). This effect is likely nonspecific, because the expression of Bax Sup. 32, an additional clone identified the same functional lethality screen as TSC22^(86)^ (C.M. Khoury & M.T. Greenwood, unpublished data), conferred a comparable resistance to the effects of *ERG6* overexpression (Fig. 5g). Finally, we examined the possibility that the prosurvival function of TSC22 is dependent on *ERG6*. Cells lacking the *ERG6* gene transformed with a TSC22^(86)^-expressing vector were significantly more resistant to the effects of H_2_O_2_ on clonogenicity with respect to control cells [TSC22^(86)^, 44.0 (±4.6) %; VECTOR, 12.4 (±3.7) %; Fig. 5h and i]. These data clearly indicate that *ERG6* is dispensable for the anti-apoptotic function of TSC-22^(86)^.

### TSC22^(86)^-mediated antiapoptosis does not require endogenous LZ-containing transcription factors

**Fig. 6:**
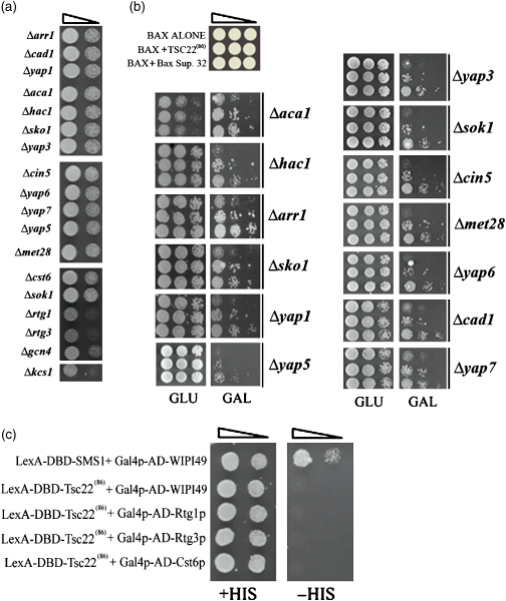
Analysis of the Bax suppressive properties of Tsc22^(86)^ in yeast mutants lacking genes encoding LZ motif-containing transcription factors. (a) Strains of yeast deleted for the indicated genes were grown overnight to saturation, serially diluted, and spotted on galactose-containing nutrient agar minimal media and incubated for 3 days at 30°C. (b) Yeast mutants lacking the indicated genes were transformed with plasmids expressing Bax alone (BAX) or in combination with plasmids expressing TSC22^(86)^ (TSC22) or Bax suppressor 32 (Bax Sup.32) were grown overnight to saturation in glucose-containing media, serially diluted and spotted onto both glucose- and galactose-containing media (GLU and GAL, respectively). A legend is shown at the top of the panel. (c) Yeast strain DSY-1 expressing Gal4 DNA-binding domain (Gal4DBD) fused to TSC22^(86)^ or SMS1 was cotransformed with different vectors encoding Gal4 activation domain (Gal4AD) fusions of the indicated LZ-containing transcription factors or WIPI49. Overnight cultures of each transformant were serially diluted, and spotted on both histidine-containing (+HIS) and histidine-deficient (−HIS) nutrient-containing agar media. The Gal4DBD-fused SMS1 and Gal4AD-fused WIPI49 pair serves as a positive control in this assay.

We used a functional genomic approach to further test the hypothesis that Tsc22^(86)^ functions by interacting with a yeast LZ-containing TF (Kester *et al.*, 1999; Uchida *et al.*, 2003). By searching the MIPS CYG database (Mewes *et al.*, 2006) for yeast genes encoding TFs with predicted leucine zippers in their structure, we identified 19 such proteins: 17 of the bzip variety possessing a basic DNA binding domain, and two LZ-containing TFs that also bear a helix–loop–helix. Many of these genes have been shown to play important roles in regulating gene expression in response to different stressful stimuli. For example, Yap1p is known to induce the expression of genes, such as heat shock proteins, that are required for survival at elevated temperatures (Rodrigues-Pousada *et al.*, 2005). Therefore, this class of proteins comprises potential candidates that interact with and mediate the effects of Tsc22^(86)^. Among the 19 genes, only one (*MET4*) has been reported to be essential (Giaever *et al.*, 2002). We initially tested the ability of strains individually lacking the remaining 18 LZ-containing TF-encoding genes to grow on minimal media containing galactose, because these conditions required to induce the expression of the TSC22^(86)^ cDNA from the *GAL1* promoter. When spotted on galactose-containing synthetic minimal media, four of the strains (Δ*rtg1*, Δ*rtg3*, Δ*gcn4*, and Δ*kcs1*) displayed moderate to severe growth defects (Fig. 6a). Deletion of one of the genes (*CST6*) resulted in only a slight impairment of growth, yet did not yield transformants with the TSC22^(86)^-expressing plasmid (not shown). There were no observable growth defects for the other deletion strains when spotted on galactose-containing media (Fig. 6a). We therefore used these strains to ask if the expression of TSC22^(86)^ retained its antiapoptotic effect in the absence of these TFs (Fig. 6b). The strains were cotransformed with different combinations of plasmids expressing BAX, TSC22^(86)^, or Sup. 32. The expression of the proapoptotic BAX protein effectively inhibited the growth of all strains examined (Fig. 6b). Upon coexpression of TSC22^(86)^ with BAX, the growth of each of the 13 strains was markedly enhanced. The effect was not specific to TSC22^(86)^, however, since Sup. 32 also served to reverse the effects of BAX in these strains (Fig. 6b). This suggests that the antiapoptotic effect of TSC22^(86)^ is independent of any individual LZ-containing TF tested. Because this experiment required the use of galactose-containing minimal media to induce gene expression and maintain plasmid selection, we were unable to test the function of TSC22^(86)^ for the five aforementioned strains displaying growth defects. As an alternate approach to determine if TSC22^(86)^ acts by forming a complex with the remaining LZ motif-containing TFs that we were unable to test, we assayed for an interaction between TSC22^(86)^ and the TFs in the two-hybrid system. To this end, we fused the TSC22^(86)^-coding region in-frame with the LexA DNA-binding domain (LexA-DBD-Tsc22^(86)^), and constructed Gal4p Activation domain fusions of each of three genes (Gal4p-AD-Cst6p, Gal4p-AD-Rtg1p, and Gal4p-AD-Rtg3p). We were unable to test Met4p, Gcn4p, or Kcs1p by the two-hybrid assay here because of difficulties generating stable plasmids with the PCR products of these genes. Neither any of the AD fusions nor LexA-DBD-Tsc22^(86)^ alone allowed for growth in the absence of exogenously added histidine, indicating that the constructs do not auto-activate the LexA-dependent *HIS3* reporter (Fig. 6c). Yeast cells co-expressing two previously known interacting proteins (LexA-DBD-SMS1α and Gal4p-AD-WIPI49) served as a positive control since they were able to grow in the absence of exogenous histidine (Yang *et al.*, 2007). Each of the AD-fusions cotransformed with LexA-DBD-TSC22^(86)^ lead to an undetectable level of growth in the absence of histidine, suggesting that these LZ-containing TFs do not interact with TSC22^(86)^ in the two-hybrid assay (Fig. 6c). The series of experiments presented above indicates that finding a LZ-mediated binding partner of TSC22^(86)^ is not a straight forward proposition and suggests that the functions of TSC22^(86)^ may not be mediated by LZ-containing TFs in yeast (Figs 5 and 6).

### The LZ motif of TSC22 is not necessary for its antiapoptotic function

**Fig. 7:**
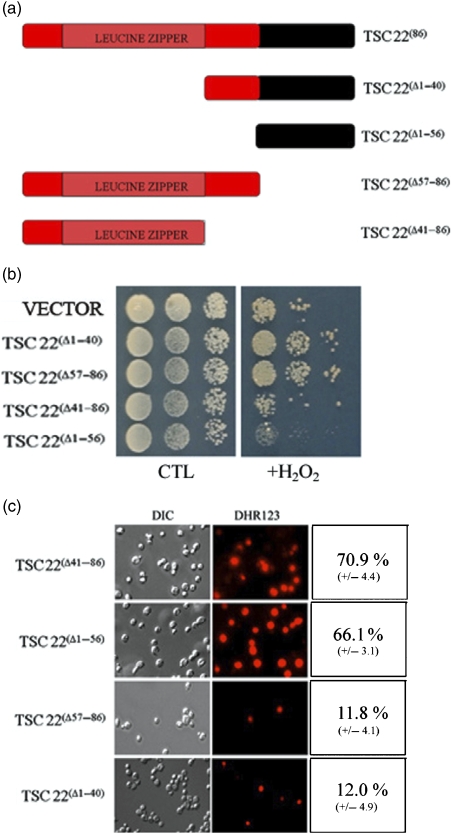
Analysis of the antiapoptotic properties of Tsc22^(86)^ deletion mutants. (a) A schematic representation of Tsc22^(86)^, as well as the deletion mutants that were generated by PCR. The PCR products were cloned into the yeast expression vector p426GAL1. (b) Wild-type yeast cells harboring empty plasmid (VECTOR) or different plasmids expressing TSC22^(86)^ deletion mutants were grown to early logarithmic phase and treated with 4 mM H_2_O_2_ for 2.5 h. Aliquots of untreated (CTL) and treated (+H_2_O_2_) cells were diluted and spotted on nutrient-containing agar media. (c) The levels of ROS were determined using the fluorescent dye DHR123 in yeast cells expressing the TSC22^(86)^ deletion mutants indicated. Representative photographs of the light (DIC) and the corresponding fluorescent image (DHR123) for the four samples are shown. The percentage of cells (±SEM) displaying a fluorescent signal was determined and is indicated in boxes at the right of rows corresponding to each of the transformants.

In order to directly test the requirement of the LZ motif of TSC22^(86)^ in the observed antiapoptotic effect, we constructed and evaluated a series TSC22^(86)^ deletion mutants. A schematic representation of the different TSC22^(86)^ mutants is shown in Fig. 7a. We used yeast transformed with these deletion mutants to assay for the retention of the antiapoptotic function of TSC22^(86)^ (Fig. 7b). As observed with TSC-22^(86)^, cells expressing either the TSC22^(Δ1−40)^ or TSC22^(Δ57−86)^ mutants were notably more resistant to the lethal effects of H_2_O_2_ as compared to control cells. In contrast, we did not observe a protective effect in cells transformed with either the TSC-22^(Δ1−56)^ or TSC-22^(Δ41−86)^ deletions. We also tested the effect of these deletion mutants on the accumulation of endogenous ROS using the ROS-activated fluorescent probe DHR123 (Fig. 7c). After treatment with H_2_O_2_, 65.9 (±4.4) % and 69.6 (±3.1) % of cells that were transformed with either TSC22^(Δ1−56)^ and TSC22^(Δ41−86)^, respectively, displayed a fluorescent signal. In cells expressing the TSC22^(Δ1−40)^ and TSC22^(Δ57−86)^ mutants the observed proportion of such ROS-positive cells was respectively 12.0 (±4.9) % and 11.8 (±4.1) %. Taken together these data demonstrate a prosurvival function for TSC22 that is independent of the LZ motif, but instead requires a 16-residue sequence located at the C-terminal portion of the conserved TSC22 domain.

### TSC22-like motif present in yeast proteins Sno1p and Fyv10p is sufficient to promote cell survival in yeast

**Fig. 8:**
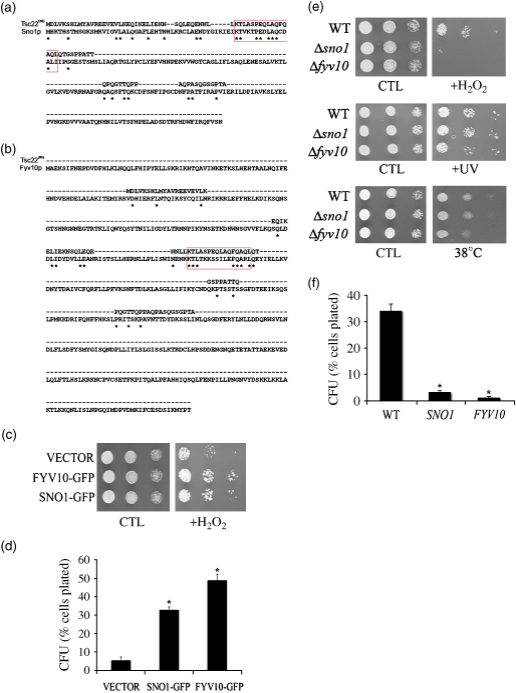
Analysis of the antiapoptotic properties of the yeast *SNO1* and *FYV10* genes an alignment of the amino acid sequence of Tsc22^(86)^ with the sequences of Sno1p (a) and Fyv10p (b). clustalw was used to align the sequences of Tsc22^(86)^ with Sno1p (GenBank accession #: NP_013813) and Fyv10p (GenBank accession #: NP_012169). The regions corresponding to the 16-residue Tsc22^(86)^ sequence responsible for its anti-apoptotic function are boxed. An asterisk (^*^) is used to indicate identical residues. Gaps denoted by dashes (‘-’) are introduced to maximize the possible similarity between the aligned sequences. (c) Wild-type cells transformed with vectors expressing GFP alone (VECTOR) or GFP fused to the C-terminus of Sno1p or Fyv10p were grown to early logarithmic phase in galactose-containing minimal media. Separate cultures of each sample were left untreated (CTL) or treated with 4 mM H_2_O_2_ (+H_2_O_2_) for 3 h. Aliquots were removed and normalized for cell number, serially diluted, then spotted on nutrient-containing agar media and incubated for 3 days. (d) Wild-type cells expressing GFP (VECTOR) with either an empty vector, or vectors encoding C-terminal GFP fusions of Sno1p or Fyv10p were grown to logarithmic phase in treated with 4 mM H_2_O_2_. Aliquots were removed at the times indicated, and an equal number of cells from each sample were spotted on nutrient-containing media and incubated for 2 days. CFU were then scored and the data expressed as ‘% of plated cells’. Data represent the mean of 3 independent experiments (±SEM). (e) Wild-type (WT), Δ*sno1*, and Δ*fyv10* cells were grown to early logarithmic phase in minimal media before cultures of each sample were left untreated (CTL) or treated with 2.5 mM H_2_O_2_ for 2 h (+H_2_O_2_). Aliquots were removed and normalized for cell number, serially diluted, then spotted on nutrient-containing agar media and incubated for 2–3 days. Untreated samples were also spotted on plates that were subsequently irradiated with UV light at 100 mJ cm^−2^ (+UV), or incubated at elevated temperature (38°C), each with corresponding untreated control plates (CTL). (F) Wild-type (WT Δ*sno1*, and Δ*fyv10* cells were grown to early logarithmic phase in minimal media before treatment with 2.5 mM H_2_O_2_ for 2 h. Aliquots were removed at the times indicated, and an equal number of cells from each sample were spotted on nutrient-containing media and incubated for 2 days. CFU were then scored and the data expressed as ‘% of plated cells’. Data represent the mean of three independent experiments (±SEM).

We examined the possibility that the 16-residue sequence required for the antiapoptotic effects of Tsc22^(86)^ in yeast represents a conserved motif present in other proteins. Upon comparison of this putative motif to the available protein sequences in GenBank, we identified a total of 19 proteins possessing similar 16-residue sequences. Among the sequences identified were four yeast proteins that were of potential interest since their antiapoptotic potential could be easily tested using the yeast assays described in the current study. Working under the hypothesis that Tsc22^(86)^ acts by mimicking an endogenous yeast molecule, we predicted that yeast proteins possessing similarity to the 16 residues necessary for the function of Tsc22^(86)^ might also be antiapoptotic. An alignment of the amino acid sequences of Sno1p (Fig. 8a) and Fyv10p (Fig. 8b) with that of Tsc22^(86)^ reveals a cluster of conserved residues corresponding to the 16-residue region required for antiapoptosis. Sequences outside this regions displayed relatively poor sequence identity. We tested the ability of two of the yeast proteins harboring the ‘Tsc22-like’ motif to promote cell survival in yeast. As shown in Fig. 8c, cells harboring plasmids encoding GFP alone displayed a reduction in viability upon treatment with H_2_O_2_ for 2.5 h. In contrast, cells expressing GFP fused with either Sno1p or Fyv10p display a marked resistance to the effects of H_2_O_2_. While only 6.0 (±0.8)% of cells harboring a vector expressing GFP alone retained the ability to form colonies after treatment with H_2_O_2_ for 2.5 h, the proportion of Sno1p-GFP and Fyv10p-GFP expressing cells remaining viable under these conditions was increased to 35.0 (±1.5%) and 48.9 (±1.6%), respectively (Fig. 8d). These results indicate that overexpression of Sno1p and Fyv10p served to significantly delay death induced by an apoptotic stimulus. If these proteins represented *bona fide* apoptotic regulators, strains lacking the genes from which they are produced are likely to be hypersensitive to the effects of H_2_O_2_. Indeed, isogenic Δ*sno1* and Δ*fyv10* cells displayed a pronounced sensitivity to the effects of H_2_O_2_ when compared to WT cells (Fig. 8e). After 2 h treatment, 35.6 (±2.8%) of WT cells retained the ability to form colonies, as compared to 3.4% (±0.7%) and 1.3 (±0.5%) for Δ*sno1* and Δ*fyv10* strains, respectively (Fig. 8f). These data indicate that the ‘TSC22-like’ motif harboring proteins Snop1p and Fyv10p are antiapoptotic.

## Discussion

Direct phenotypic screens for antiapoptotic proteins in which clones are identified on the basis that they prevent cell death in response to a specific stimulus are technically difficult in most metazoan systems. In contrast, forward genetic screens in simple genetic systems, such as *C. elegans* and *Drosophila melanogaster*, have been extensively performed in the past. Such screens have lead to the identification of many conserved apoptotic regulatory molecules, including the central proapoptotic caspase-3, caspase-9, and Apaf-1 (Hoeppner *et al.*, 2004). More recently, several groups have exploited the amenability of yeast to functional screens to identify novel heterologous proteins capable of suppressing the lethal effects of apoptotic stimuli (Kampranis *et al.*, 2000; Pan *et al.*, 2001; Moon *et al.*, 2002; Sawada *et al.*, 2003; Chen *et al.*, 2004; Yang *et al.*, 2006; Khoury *et al.*, 2007; Khoury & Greenwood, 2008). The human bax-inhibitor I (BI-I) protein, for instance, was initially isolated in a screen for suppressors of Bax in yeast, and has since been shown to be an antiapoptotic factor conserved across plant, animal, and fungal kingdoms (Xu & Reed, 1998). Sawada *et al.* (2003) identified mammalian Ku70, a protein previously implicated in DNA damage repair, in a screen for suppressors of the effects of Bax in yeast. Ku70 was found to bind Bax and prevent its translocation to the mitochondria, thereby serving as a specific inhibitor of Bax-mediated apoptosis. Additional heterologous suppressors of apoptotic stimuli identified in yeast-based screens included the tomato glutathione peroxide, the soybean ascorbate peroxidase, and both a Vesicle-Associated Membrane Protein (VAMP7) and the Ethylene-Responsive-Element Binding Protein (AtEBP) from *Arabidopsis*, the murine sphingomyelin synthase 1α (Sms1α), and human Vacuolar protein sorting 24β (VPS24β) (Kampranis *et al.*, 2000; Pan *et al.*, 2001; Moon *et al.*, 2002; Sawada *et al.*, 2003; Chen *et al.*, 2004; Yang *et al.*, 2006; Khoury *et al.*, 2007). Therefore, screening heterologous cDNA libraries in yeast has proven to be an excellent approach to identify novel antiapoptotic sequences.

In this study, we identify the 86 C-terminal residues of the Tsc22 protein (denoted Tsc22^(86)^) as a high-copy suppressor of the deleterious effects of murine BAX expression in yeast (Fig. 1). Tsc22^(86)^ represents the C-terminal TSC22-containing sequence encoded by the *TSC22-1* gene and contains the highly conserved 56 aa LZ motif-containing domain known as TSC22 (Figs 1b and 2a) (Kester *et al.*, 1999). Analysis of this domain revealed that it is present in multiple proteins of different sizes. We used the various TSC22-containing proteins present in the GenBank database to determine that there are four different alternately spliced human genes that encode for a TSC22 motif (Figs 2 and 3). We confirmed an antiapoptotic role for the Tsc22^(86)^ by demonstrating that it attenuated Bax- and H_2_O_2_-mediated cell death (Fig. 4a and 4b), as well as death induced by yeast caspase *YCA1* overexpression (Fig. 4c). We additionally showed that Tsc22^(86)^ prevented H_2_O_2_-induced intracellular ROS accumulation, a hallmark of apoptotic cells (Fig. 4d). Tsc22^(86)^ therefore represents the most recent member in a growing list of both endogenous and heterologous proteins that have been shown to prevent apoptotic cell death in yeast (Walter *et al.*, 2006).

### The TSC22 domain defines a multi-gene family each encoding different proteins

The evolution of gene families is the result of different processes such as duplication of regions of genes, whole genes, or entire genomes and the recombination of protein-coding sequences (Babushok *et al.*, 2007). These phenomena form the basis for the evolution of genetic diversity. The divergences that can be observed between paralogous genes (as a result of duplication events) or the generation of novel roles for genes that have gained an additional functional domain (as a result of exon shuffling) are extensively documented outcomes of these events (Schmidt & Davies, 2007). For instance, a genome duplication event in a close ancestor of *S. cerevisiae* is thought to have occurred, based in part on the observation that more than 40% of its genes exist as paralogous pairs (Wolfe, 2004). Alternatively, Liu *et al.* compared protein domain architecture to exon-intron organization of genes across nine eukaryotic genomes (Liu & Grigoriev, 2004). Their finding indicated a strong correlation between the borders of protein domains and the ends of exons and that this correlation increased with progressive organismal complexity, providing evidence for the theory of exon shuffling. We found that with regard to *TSC22-1*, *TSC22-3* and *TSC22-4*, their sequence identity is limited to their common TSC22 domain-encoding regions (Figs 2 and 3). The presence of the TSC22 domain within the human *TSC22* gene family is likely to be the result of exon shuffling events, with dissimilar pairs of genes being ‘converted’ by gaining the TSC22 domain. The conserved TSC22 domain is indeed specified by a single exon in all four human and mouse genes and therefore appears to be in accordance with the principles of the exon shuffling theory (Figs 2 and 3) (Liu & Grigoriev, 2004; Fiol *et al.*, 2007). On the other hand, *TSC22-1* and *TSC22-2* share significant sequence identity outside the TSC22 domain, suggesting that these genes arose by duplication. Diversity involving both duplication and exon shuffling within the same gene family is commonly observed (Jean-Baptiste *et al.*, 2006).

More than half of human genes are thought to encode alternately spliced products, suggesting the major importance of this mechanism in generating diversity within the proteome (Johnson *et al.*, 2003). It is widely recognized that the proportion of different isoforms encoded by the same gene is often altered in certain pathological states such as cancer (Venables, 2006). Often a spliced transcript that is selectively produced in pathological or stressful conditions encodes for a protein with a different or regulatory function with respect to the transcript expressed predominantly in basal conditions (Venables, 2006). The significance of this regulatory mechanism is clearly evident with regard to genes involved in apoptosis. For instance, the BCL-X pre-mRNA is alternatively spliced to produce both pro- (Bcl-xS) and antiapoptotic (Bcl-xL) isoforms. In addition to being markedly upregulated in a number of tumors, the expression of Bcl-xL is reduced (concomitant with an upregulation of Bcl-xS) in cells treated with apoptotic stimuli (Boise *et al.*, 1993). As in this case, the selective up- or down-regulation of a given isoform through alternative splicing is often an important determinant of cell survival (Venables, 2006). We found that in addition to our TSC22^(86)^ clone, the N-terminally extended forms of the Tsc22-1 proteins are also antiapoptotic when expressed in yeast (Fig. 2c). Separate assays will be required in order to uncover the function of the alternatively spliced variants of the *TSC22* genes. In this context, it is interesting to note that the *TSC22-4* gene encodes for a protein (Tsc-22-4v2) that lacks the antiapoptotic TSC22 domain (Fig. 3c and f), suggesting a possible dominant negative mode of action in cells coexpressing different Tsc22-4 proteins. In addition, the observation that a number of transcripts from different TSC22 genes are upregulated in response to a variety of different stimuli suggests that they may have a role to play in mediating stress responses (Uchida *et al.*, 2003; Fiol *et al.*, 2007).

### TSC22 is antiapoptotic independently of the LZ motif in yeast

TSC22 domain-containing proteins are reported to have effects on transcription by virtue of an embedded LZ motif. Further, they are thought to be transcriptional modulators, rather than direct TFs, due to the lack of any apparent DNA-binding domain (Kawamata *et al.*, 2004). It has been proposed that Tsc22 functions through the heterodimerization with cognate LZ motif-containing transcriptional regulators through an LZ mediated process (Kawamata *et al.*, 2004). This would likely involve either a gain-of-function or a dominant inhibitory interaction between Tsc22 and the putative LZ-containing protein. Similar paradigms have been documented in the study of other LZ-containing molecules (Vinson *et al.*, 2002; Rangatia *et al.*, 2003; Thuerauf *et al.*, 2004; Benito *et al.*, 2006; Gonzalez *et al.*, 2007). For instance, the LZ-containing transcriptional activators c-jun and C/EBP-α have opposing effects on cell proliferation. In a recent model of acute myeloid leukemia, c-jun was shown to act by binding in an LZ-mediated manner to the antiproliferative C/EBP-α protein. This dominant negative inhibition of C/EBP-α prevented its ability to bind DNA and thereby promote oncogenesis (Rangatia *et al.*, 2003). Another example of this mechanism pertaining to the regulation of apoptosis involves thyrotroph embryonic factor (TEF) and D-site-binding protein (DBP), members of the proline- and acid-rich (PAR) basic region LZ (bzip) proteins (Benito *et al.*, 2006). TEF acts by forming LZ-mediated homodimers that bind to the promoter region to active the expression of the proapoptotic Bcl-gS protein. An alternately spliced form of DBP (tDBP) that lacks the transcriptional activation domain was shown to prevent activation of the BCL-gS gene by competing with TEF monomers and impairing the formation of a functional heterodimer in a manner mediated by the LZ structure (Benito *et al.*, 2006). Based on the aforementioned descriptions of the mechanisms by which Tsc22 has been proposed to function, we used global yeast two-hybrid screening and functional genomics, two separate and distinct approaches to test this model for Tsc22^(86)^ in yeast.

In contrast to conventional cDNA library-based yeast two-hybrid screens, the development of genome-wide screening technologies have allowed comprehensive and exhaustive screening for bait-interacting proteins. Uetz *et al.* constructed an array of yeast transformants expressing all of the recognized *S. cerevisiae* ORFs fused to a transcriptional activation domain (Uetz *et al.*, 2000). By mating these strains with a strain expressing a bait-DNA-binding domain fusion, an interaction between any desired protein and the entire yeast proteome can be assayed in an automated, binary fashion. For example, the yeast regulator of G-protein signaling (RGS) Sst2p was used as bait in a genome-wide two-hybrid screen that yielded 17 interacting proteins. This resulted in the identification of novel and critical components of the Sst2p-regulated signaling pathways as evidence by the altered pheromone response of strains lacking these genes (Burchett *et al.*, 2002). Previous groups have been successful in identifying LZ-mediated interactions using the yeast two-hybrid system (Strathmann *et al.*, 2001; Weltmeier *et al.*, 2006). Yeast two-hybrid screening for Tsc22-interacting proteins identified the LZ motif-containing protein encoded by the *TSC22-4* gene as a binding partner of Tsc22 (Kester *et al.*, 1999). While, in the current study, a genome-wide two-hybrid analysis revealed an interaction between Tsc22^(86)^ and two yeast proteins, Nkp1p and Erg6p, we were surprised that neither of these possessed a LZ structural motif as predicted by the analysis of their primary sequences. Although these proteins may indeed represent heterologous Tsc22^(86)^-interacting partners, we clearly demonstrated that their presence is not required for the antiapoptotic function of Tsc22^(86)^ (Fig. 5e, f, i and j)

Functional genomics approaches have been widely used in studies using *S. cerevisiae*, due to the extensive resources available and genetic amenability of this model system. In a recent report, a set of strains harbouring deletions in previously uncharacterized small ORFs (sORFs) was constructed. This study provided an array of phenotypic descriptions for a number of strains lacking these sORFs, including information on growth rates, heat-shock response, responses to DNA damaging agents, and growth under respiratory conditions (Kastenmayer *et al.*, 2006). Many of these sORFs were also shown to be conserved across eukaryotes (Kastenmayer *et al.*, 2006). Therefore, the functional genomic analysis served to reveal biological functions for an important class of genomic elements. While studying the phospho-regulation of the amphiphysin yeast ortholog Rvs167p, the Andrews group screened a panel of yeast deletion strains to discover that a nonphosphorylatable mutant form of this protein prompted lethality in a subset of strains displaying defects in the actin cytoskeleton (Friesen *et al.*, 2003). This observation indicated that Rvs167p phosphorylation is a key event in the regulation of actin cytoskeleton-associated complexes (Friesen *et al.*, 2003). Finally, the dependency that Bax-mediated cell death displays on the respiratory status of yeast was demonstrated by examining the function of Bax in a set of mutant yeast strains with specific respiratory-defects (Harris *et al.*, 2000). By further demonstrating that expression of the heterologous Bax protein causes respiratory dysfunction in yeast, this study bulwarked the notion that Bcl-2 family members can act independently (the yeast genome is devoid of any apparent *BCL-2* orthologs) to alter mitochondrial physiology (Harris *et al.*, 2000). Our functional genomic analysis of Tsc22^(86)^ in strains lacking genes that encode LZ-containing TFs comprises the first description of the collective panel of strains deleted for LZ-containing TFs (Fig. 6). A number of these proteins have been extensively implicated in the regulation of stress responses in yeast and therefore likely to be involved in the apoptotic programme. For instance, the expression of a number of genes encoding the LZ-containing TFs, such as *YAP4* and *YAP6*, are induced by a number of apoptotic stimuli, including heat, osmotic and oxidative stresses (Rodrigues-Pousada *et al.*, 2005). In addition, the stress-induced expression of genes encoding a number of antioxidant proteins, including *TRX2*, is dependent on the bzip TF Yap1p (Alarco & Raymond, 1999). Kilili *et al.* (2004) identified a class of tomato Glutathione *S*-transferases that suppress oxidative stress-induced cell death in yeast. This protective effect was abrogated in strains lacking the *YAP1* gene, providing further evidence for the role of Yap1 in oxidative stress responses. Our results suggest that the antiapoptotic function of Tsc22^(86)^ is not dependent on any individual LZ-containing yeast TFs (Fig. 6). This is consistent with the lack of LZ-containing proteins in our findings from the global two-hybrid screen (Fig. 5).

Structure/function studies using deletion mutants of Tsc22-1v3 have attributed roles to both the conserved and nonconserved regions of the protein. For instance, mutants of Tsc22-1v3 deleted for two independent regions at flanking ends of the conserved TSC22 domain displayed a reduced ability to act as a repressor in a reported-based transcriptional activity assay in COS-I cells. This allowed for the identification of two repressor domains (RD1 and RD2) in the nonconserved portions of the Tsc22-1v3 protein (Kester *et al.*, 1999). Hino *et al.* (2002) demonstrated that overexpression of the LZ domain from the same Tsc22 protein resulted in a more pronounced inhibition of anchorage-independent colony formation in a salivary gland cancer cell and CHO cell lines when compared to the full length protein. These results suggest that the LZ structure of Tsc22-1v3 is a functional domain that serves to suppress tumour cell growth. More recently, the expression of a mutant lacking the N-terminal 26 residues of the TSC22 domain (yet retaining the LZ motif) failed to promote the growth-inhibitory effect observed upon expression of full-length Tsc22 in *Xenopus laevis* cells (Hashiguchi *et al.*, 2007). Therefore, there is evidence for both LZ motif-dependent and independent functions for Tsc22-1v3 in the literature. Our finding that deletion mutants lacking the LZ motif retain the antiapoptotic function clearly demonstrates a LZ-independent function for Tsc22^(86)^ in yeast. In effect, our results suggest that a 16 aa stretch C-terminal to the LZ motif is necessary for the prosurvival effect. Our findings demonstrate the requirement of a previously unrecognized region in the pro-survival function of Tsc22^(86)^.

### The ‘Tsc-22-like’ motif predicts an antiapoptotic role for SNO1 and FYV10

Deletion analyses have been tremendously successful at identifying functional domains for a large number of different genes (Reed *et al.*, 1996). Examples include genes encoding the Bcl-2 homology domains (BH1–BH4), present in the Bcl-2 family of pro- and antiapoptotic proteins (Reed *et al.*, 1996). BH domains are comprised of short sequences that form common tertiary structures such as α helices. Their importance is demonstrated by the finding that deletion of any of the BH domains of Bcl-2, including the BH1 (28 aa), BH2 (15 aa), or BH3 (13 aa) abrogates its ability to mediate antiapoptosis (Reed *et al.*, 1996). Importantly, the presence of BH domains in other proteins has allowed the identification of other Bcl-2 family members with roles in the regulation of apoptosis (Adams & Cory, 1998). In the current study, we have identified a 16-residue sequence, comparable in length to the BH domains, within the conserved TSC22 domain that is required for the antiapoptotic effect of Tsc22^(86)^ in yeast. In a manner analogous to the aforementioned BH domain studies, we used blast to identify 19 different proteins that contain a sequence similar to the antiapoptotic TSC22 motif. Thus, we predicted that these ‘TSC22-like’ proteins might represent a novel class of antiapoptotic sequences. We tested this prediction by analyzing two of the four yeast genes identified, namely *SNO1* and *FYV10* (Fig. 8). Both genes were found to be antiapoptotic since their overexpression protected cells from H_2_O_2_ (Fig. 8c and d). Additionally, strains lacking these genes were considerably more sensitive to the effects of H_2_O_2_ (Fig. 8e and d). Thus, our characterization of Tsc22^(86)^ in yeast has served to functionally identify novel antiapoptotic genes. Based on our data, other proteins possessing the Tsc22-like motif are strong candidates for proteins with antiapoptotic function.

*SNO1* was originally identified on the basis of its proximity to *SNZ1*, a related gene with which it is coordinately regulated in a growth-phase dependent manner. For instance, both genes are upregulated during the diauxic shift that occurs upon entrance into stationary phase, and have been proposed to be involved in the adaptive response to nutrient limitation (Padilla *et al.*, 1998). In addition, Sno1p has been reported to function as a glutaminase in a manner dependent on Snz1p, serving to promote pyridoxine biosynthesis (Rodriguez-Navarro *et al.*, 2002; Dong *et al.*, 2004). The ability of Sno1p to protect from oxidative stresses may be simply due to an elevation in the levels of vitamin B6, a molecule with known ROS scavenging functions (Osmani *et al.*, 1999). The deletion of *SNO1* had been previously reported to result in hypersensitivity to inhibitors of purine and pyrimidine biosynthesis, as well as ROS-generating agents (Padilla *et al.*, 1998). In yeast, *SNO1* is part of a multigene family that includes *SNO2* and *SNO3* (Padilla *et al.*, 1998). Although the 222 residue Sno2p and Sno3p differ in only two residues, they are only 65% identical to the 224 residue Sno1p. Of the 16 TSC22 motif present in Sno1p, only 11 are conserved in Sno2p and Sno3p. In addition, to differences in sequences and differences in their regulation, *Sno2* and *Sno3* double knockouts, unlike Δ*sno1* strains, have normal sensitivity to inhibitors of purine and pyrimidine biosynthesis. Thus they appear to be less likely to be involved in stress response.

*FYV10* was identified in a screen for mutants hypersensitive to the death inducing effects of viral killer toxin K1 (Page *et al.*, 2003). Although this study is suggestive of a role for *FYV10* in the regulation of apoptosis, it should be noted that a great number of genes have been identified as conferring increased sensitivity to a variety of stresses when they are knocked-out (Page *et al.*, 2003; Scherens & Goffeau, 2004). Despite these phenotypes, only a subset of these genes is expected to confer resistance to these stresses when overexpressed, and therefore be *bona fide* anti-apoptotic regulators (Khoury *et al.*, 2007). Our study is the first to clearly demonstrate the importance of *FYV10* in preventing death in response to apoptotic stimuli.

It is worth noting that Fyv10p possesses a conserved CTLH domain, within which is the 16-residue ‘Tsc22-like’ sequence. There are four yeast proteins with CTLH domains (Regelmann *et al.*, 2003). All four are encoded by the so-called *GID* genes, defined by a function in the proteosome-dependent glucose-induced catabolite degradation of the gluconeogenic enzyme fructose-1,5-bisphosphatase (Regelmann *et al.*, 2003). The potential role of these proteins in regulating apoptosis is at present unknown. Interestingly, the human erythroblast macrophage protein (Emp) shares 22–30% identity to Fyv10p throughout much of its sequence. While Emp has been suggested to perform an antiapoptotic function, the mechanistic details are currently unknown (Hanspal *et al.*, 1998).

## Conclusion

The use of yeast as a heterologous system to dissect the structure and function of mammalian genes has a long history (Hartwell, 2002). Functional analysis in yeast of mutant Bax lacking critical residues within the BH3 domain pointed to the importance of this domain in Bax-mediated apoptosis in mammalian cells (Zha *et al.*, 1996). Although yeast has been used to study metazoan proteins involved in apoptosis, our study is one of the first to demonstrate that the similarity between mammalian and yeast apoptosis allows the detailed analysis of a heterologous apoptotic regulator. The current study also provides a clear illustration of the insight that can be gained through such an analysis.
